# Early introduction of exercise prevents insulin resistance in postnatal overfed rats

**DOI:** 10.1590/1414-431X2022e11987

**Published:** 2022-07-13

**Authors:** S.V. Fischer, M.H. Appel, K. Naliwaiko, D.D. Pagliosa, D.N. Araújo, A.E. Capote, B.A.C. Oliveira, L.C. Fernandes

**Affiliations:** 1 Departamento de Fisiologia, Setor de Ciências Biológicas, Universidade Federal do Paraná, Curitiba, PR, Brasil; 2 Departamento de Biologia Estrutural, Molecular e Genética, Universidade Estadual de Ponta Grossa, Ponta Grossa, PR, Brasil; 3 Departamento de Biologia Celular, Setor de Ciências Biológicas, Universidade Federal do Paraná, Curitiba, PR, Brasil

**Keywords:** Postnatal overnutrition, Metabolic programming, Exercise, Muscle metabolism, Adipose tissue

## Abstract

Early childhood obesity increases the risk of developing metabolic diseases. We examined the early introduction of exercise in small-litter obese-induced rats (SL) on glucose metabolism in the epididymal adipose tissue (AT) and soleus muscle (SM). On day 3 post-birth, pups were divided into groups of ten or three (SL). On day 22, rats were split into sedentary (S and SLS) and exercise (E and SLE) groups. The rats swam three times/week carrying a load for 30 min. In the first week, they swam without a load; in the 2nd week, they carried a load equivalent to 2% of their body weight; from the 3rd week to the final week, they carried a 5% body load. At 85 days of age, an insulin tolerance test was performed in some rats. At 90 days of age, rats were killed, and blood was harvested for plasma glucose, cholesterol, and triacylglycerol measurements. Mesenteric, epididymal, retroperitoneal, and brown adipose tissues were removed and weighed. SM and AT were incubated in the Krebs-Ringer bicarbonate buffer, 5.5 mM glucose for 1 h with or without 10 mU/mL insulin. Comparison between the groups was performed by 3-way ANOVA followed by the Tukey *post-hoc* test. Sedentary, overfed rats had greater body mass, more visceral fat, lower lactate production, and insulin resistance. Early introduction of exercise reduced plasma cholesterol and contained the deposition of white adipose tissue and insulin resistance. In conclusion, the early introduction of exercise prevents the effects of obesity on glucose metabolism in adulthood in this rat model.

## Introduction

Early childhood obesity increases the risk of metabolic diseases such as insulin resistance (IR), type 2 diabetes (T2D), cardiovascular disease, and other metabolic disorders (1). In humans, the prevalence of obesity in children has increased markedly (2).

Nutrition during fetal life and soon after birth (metabolic programming) may predispose individuals to metabolic dysfunctions such as obesity (3). This programming may cause disturbances, which might be passed on to future generations (1). Moreover, changes in the environment and energy supply can affect development (4).

Plagemann et al. developed an experimental model of obese rodents by raising rats in small litters (5), which reduces competition for the mother's milk during the lactating period and, therefore, leads to overnourishment because the total calorie intake for each pup is increased. In this model, there are nutritional disturbances during the breastfeeding period that can lead to the development of short- and long-term changes not only in body weight but also in the white adipose tissue (AT) (1). In addition, the rats show an accelerated body weight gain and a long-lasting obese phenotype associated with permanent modulation of hypothalamic circuits that control food intake and energy balance in adulthood (6).

Skeletal muscle (SM), the main site of insulin-dependent glucose disposal, takes up more than 80% of an intravenous glucose load (7). A signal from insulin results in translocation of glucose transporter-4 (GLUT4) to the cell membrane (8). A failure in insulin action leads to insulin resistance (9). Animals that are overnourished during lactation have an epigenetic modification in the insulin-signaling pathway (10). In obese rats, insulin resistance is related to alterations in the expression of insulin receptor substrate-1 (IRS-1) and reduction in the expression of GLUT4, PI3K, and Akt (9,10).

The prevalence of obesity in children has escalated markedly worldwide (11). Inactivity during childhood affects physical well-being in children (12). Exercise is an effective non-pharmacological strategy, promoting well-being and reducing the risk of certain diseases due to the induction of several adaptations at the metabolic level by modulating the expression of specific genes (13). Indeed, in obese animal models and humans, exercise increases insulin sensitivity and augments glucose transport by a non-insulin-dependent pathway (14).

In the present work, we aimed to examine whether early introduction of exercise in 22-day-old small-litter rats prevents in adulthood the effect of obesity on glucose metabolism *in vivo* and *in vitro*. For this purpose, we determined several morphometric and biochemical parameters in four groups: sedentary, exercise, small-litter sedentary, and small-litter exercise rats.

## Material and Methods

Unless otherwise indicated, chemicals and enzymes were purchased from Sigma Chemical Co. (USA). Radiochemicals were obtained from NEN Life Sciences Products, Inc. (USA). Humulin^®^ R U-100 insulin was obtained from Eli Lilly and Company (USA). Assay kits for measurements of biochemical plasma parameters were purchased from Laborclin (Brazil).

### Animals

All animal protocols were approved by the Ethics Committee for Experimental Animals from the Federal University of Paraná (CEUA number 865/2015). Animals were housed under a 12-h light/dark cycle at 23±1°C with free access to water and chow. The animals received a regular chow diet (protein content 230 g/kg, vitamins 660 g/kg, fat 40 g/kg, fiber 60 g/kg, and minerals 10 g/kg; Nuvital^®^, Brazil).

Food and body weight were monitored every two days from weaning until the age of 90 days.

### Induction of obesity by metabolic programming

A total of 21 dams (age of 90 days) were used, of which sixteen were separated for small litter (SL) and five were used as control. After birth, each dam had ten pups. On day three post-birth, one group was kept with three pups per dam, resulting in early overfeeding during lactation (5). The other group was kept with 10 pups per dam. The pups were weaned at 21 days of age (3 male rats per cage). Then, each group was subdivided into two experimental groups forming 4 groups: sedentary (S), exercise (E), small-litter sedentary (SLS), and small-litter exercise (SLE).

### Swimming training

After weaning, swimming training was performed as described in our previous work (15). The swimming system consisted of a central compartment and 10 tanks (250 mm diameter and 460 mm high, with 330 mm water level) for individual swimming to avoid the effects of crowding on performance. Briefly, from 22 to 90 days of age, the rats swam three times/week for 30 min. In the first week, they swam without a load; in the 2nd week, they carried a load equivalent to 2% of their body weight; from the 3rd to the last week, they carried a 5% body load attached to their tails. Water temperature was 32±2°C. The use of different load intensities is common in swimming protocols for rats. Slowly increasing the load from 2 to 5% promotes smooth training adaptation while maintaining a low stress level (16). We admit that it is quite difficult to control training intensity when weight is applied to the tail.

### Intraperitoneal insulin tolerance test

For the intraperitoneal insulin tolerance test (ITT), we selected some rats from each group at 85 days of age. The ITT was done in 12-h fasting rats 48 h after the last swimming session. Insulin (Humulin^®^ R U-100) 1 U/kg was administered intraperitoneally. Blood samples were collected at 0, 5, 8, 10, 12, 16, 20, and 25 min for glucose determination (LifeScan One Touch, Brazil).

### Blood biochemical measurements

Rats not submitted to the ITT were killed at 90 days of age. All experimental protocols were performed 48 h after the last swimming session to avoid any potential acute effect of exercise. After 12 h of fasting, rats were killed by decapitation and total blood was collected in heparinized tubes. Plasma concentrations of glucose, cholesterol, and triacylglycerol were measured by enzymatic procedures using commercial kits.

### Adipose tissue harvesting

Fat tissues from mesenteric, epididymal, retroperitoneal, and brown tissues were rapidly removed, and their weight was measured on a digital scale (Denver Instruments Company AA-200, USA).

### Incubation of soleus muscle and epididymal fat

Soleus muscles from each leg were rapidly and carefully isolated, split longitudinally into two equal portions (20-30 mg), and attached through the tendons to a small clip to keep the fibers extended. Then, muscles were pre-incubated for 30 min in Krebs-Ringer bicarbonate buffer pre-gassed for 30 min with 95% O_2_/5% CO_2_ at 37°C containing 5.6 mM glucose, 1.5% BSA, pH 7.4. Then, the muscles were transferred to flasks that contained identical buffer plus 0.1 µCi/mL D-[U-^14^C]-glucose in the absence or presence of insulin (Humulin^®^ R U-100, 10 mU/mL). After incubation for 60 min, muscles were removed, and the incubation medium was frozen for lactate measurement (17).

Epididymal fat pads (20-30 mg) were minced in very small pieces using scissors. They were then incubated in Krebs-Ringer bicarbonate buffer at 37°C containing 5.6 mM glucose, 1.5% BSA, pH 7.4, plus 0.1 µCi/mL D-[U-^14^C]-glucose in the absence or presence of insulin (10 mU/mL). After incubation for 60 min, we added perchloric acid (25%) to stop the reaction. The medium was collected and neutralized with Tris-KOH (2-0.5 M). The medium was harvested for total and radiochemical lactate production measurement.

### Lactate production measurement

Total lactate production by isolated incubated soleus muscles and epididymal fat tissues was assayed by measuring absorbance at 340 nm (spectrophotometric Infinite^®^ 200 Pro, USA, TECAN series) as described elsewhere (17). It is important to note that the net rate of lactate formation, which is measured by a spectrophotometric assay, is a measure of the rate of glycolysis from glucose, which can come from either muscle glycogen and/or the incubation medium. Radiochemical lactate was determined as described elsewhere (18).

### [^3^H]-2-deoxy-D-glucose uptake

Sugar transport activity was measured using the non-metabolizable glucose analog [^3^H]-2-deoxy-D-glucose (19). Briefly, incubation was done as described above but in the presence of [^3^H]-2-deoxy-D-glucose. After incubation, the muscles were frozen in liquid nitrogen, weighed, and digested for 15 min at 70°C in KOH (1 M). Muscle aliquots were placed in vials with scintillation fluid and glucose was measured in the Beckman LS 6000 IC equipment (USA).

### Statistical analyses

Data are reported as means±SE. The data were evaluated for normality (Shapiro-Wilk test) and homoscedasticity (Bartlett test). Ordinary two-way ANOVA was used for fat tissue weight and blood biochemical parameters (factors: litter size and exercise). Two way ANOVA with repeated measures was used for ITT, body weight, and food intake (factors: litter size × exercise × time), followed by Tukey's *post hoc* test. Three-way ANOVA was used for glucose uptake and lactate production (factors: litter size × exercise × insulin) followed by Sidak's *post hoc* test. P<0.05 was used to indicate statistical significance. Statistical tests were performed using GraphPad Prism version 8.0 for Windows (GraphPad Software, USA).

## Results

**Figure 1:**
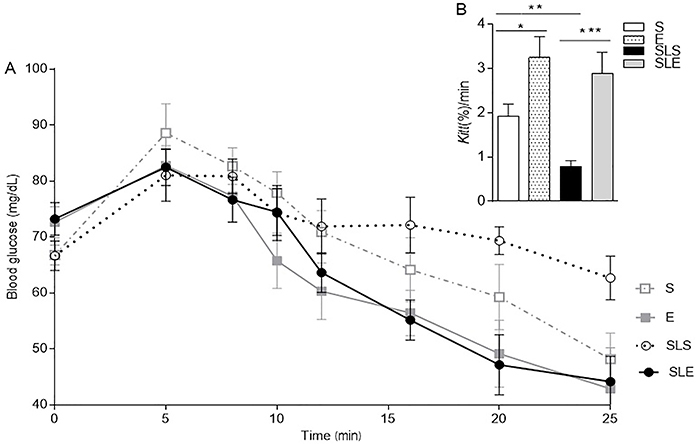
Insulin tolerance test (1.0 U/kg) at 85 days of age (**A**) in the sedentary (S), exercise (E), small-litter sedentary (SLS), and small-litter exercise (SLE) groups. **B**, Glucose disappearance rate (*Kitt*) of each group. Data are reported as means±SE (n=8-10). *P<0.05; **P<0.005; ***P<0.001 (ANOVA).

**Figure 2:**
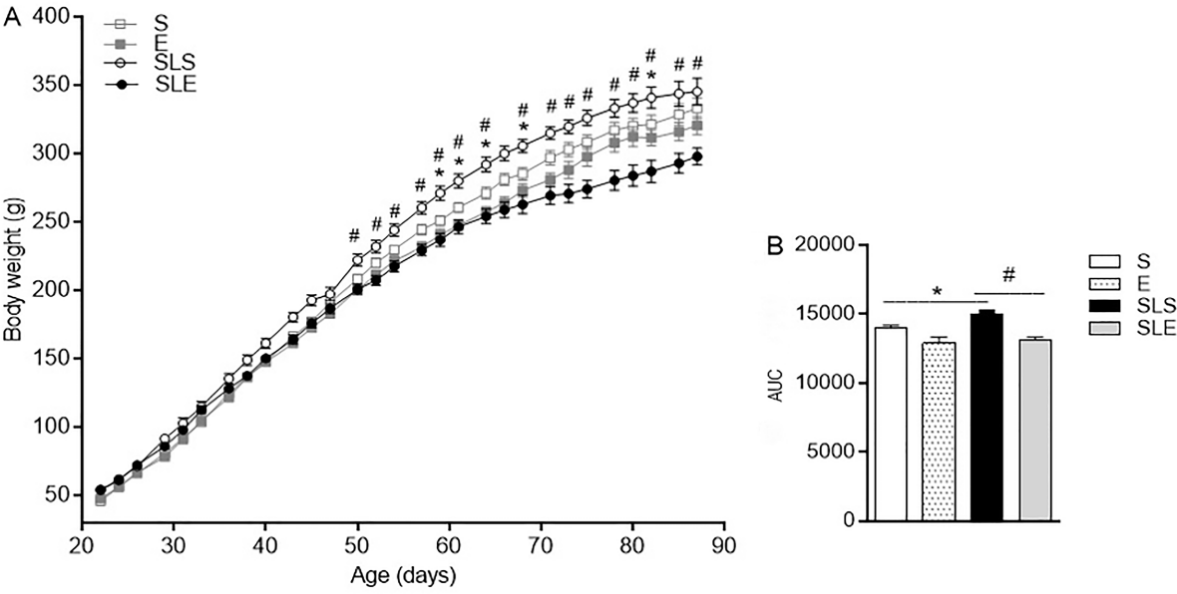
Body weight from sedentary (S, n=10), exercise (E, n=11), small-litter sedentary (SLS, n=14), and small-litter exercise (SLE, n=18) rats measured from 22 to 90 days of age (**A**). Body weight (**B**) evaluated by area under the curve (AUC). Data are reported as means±SE. ^#^P<0.05, SLS *vs* SLE; *P<0.05, S *vs* E and SLS (ANOVA).

At 85 days of age, some rats were subjected to an insulin tolerance test (ITT). In the small-litter sedentary (SLS) group, from 15 min to the end of the ITT, blood glucose concentration was higher compared to the other groups (Figure 1A). Exercise (E) (n=10) increased insulin sensitivity markedly (Figure 1B) by 95.7% compared to the sedentary (S, n=8) group (P<0.0402). SLS (n=10) had higher body weight (Figure 2A and B) and an increased insulin resistance of 52.3% compared to the S group (P<0.0027). On the other hand, the early introduction of exercise in the small-litter exercise group (SLE, n=10) did avoid insulin resistance compared to the SLS group (P<0.0014). No statistical difference was found between exercise groups (P=0.59, E *vs* SLE).

The body weight of all groups increased with age (Figure 2A). The SLS group (n=14) had significantly higher body weight from 50 to 90 days compared to the S (P=0.036), E (P=0.0006), and SLE groups (P=0.0001). The E group (n=11) had a lower body weight but was not different compared to the S group (P>0.05). The early introduction of exercise caused a similar body weight gain in the SLE group (n=18) and E group (P>0.05), which was significantly lower compared to the SLS rats (P<0.05). From 22 to 90 days of age (Figure 2B), the area under the curve (AUC) was 7.4% greater in the SLS rats compared to S rats (P<0.05). On the other hand, in the SLE, the AUC was 12.4% smaller compared to the SLS rats (P<0.05). No significant difference was found between the S and E groups (P>0.05).

**Figure 3:**
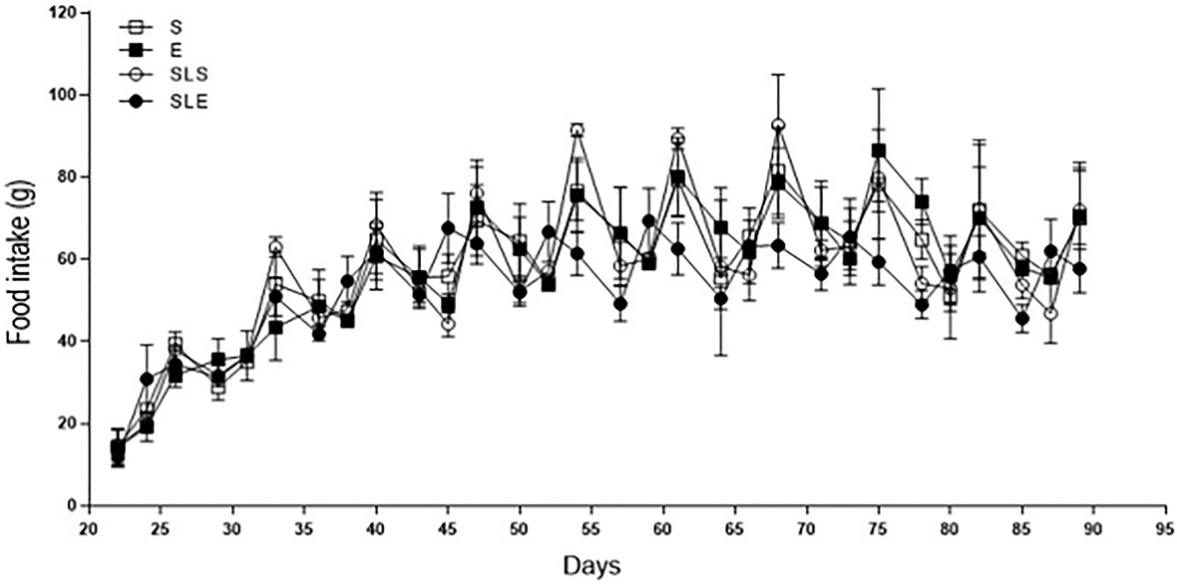
Food intake (g) in sedentary (S, n=10), exercise (E, n=11), small-litter sedentary (SLS, n=14), and small-litter exercise (SLE, n=18) rats measured from 22 to 90 days of age. Data are reported as means±SE of at least 10 rats per group. P>0.05 (ANOVA).

From 22 to 90 days of age, no statistical difference in food intake was seen between groups (P>0.05, Figure 3).

**Figure 4:**
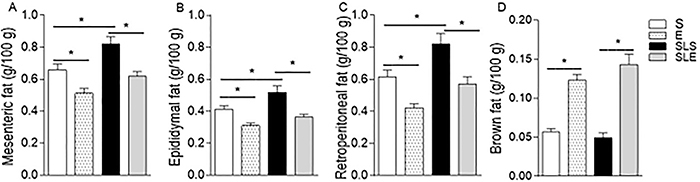
Weight of mesenteric (**A**), epididymal (**B**), retroperitoneal (**C**), and brown (**D**) fat tissues from sedentary (S), exercise (E), small-litter sedentary (SLS), and small-litter exercise (SLE) groups. Data are reported as means±SE of 10-18 rats per group. *P<0.05 (ANOVA).

In the SLS group, the weight increased by 24% in the mesenteric (Figure 4A), 26% in the epididymal (Figure 4B), and 32% in the retroperitoneal (Figure 4C) fat pads compared to the S group (P<0.05). In both exercise groups (E and SLE), white fat tissues had a lower weight compared with non-exercise groups (S and SLS). In the E and SLE groups, mesenteric fat weight was 22.8 and 24.4%, epididymal fat was 24.4 and 30.7%, and retroperitoneal fat was 32.3 and 30.5% lower, respectively, compared to the S groups (P<0.05). The early introduction of exercise (E and SLE) increased brown fat weight by 118 and 187% compared to non-exercise groups (P<0.05, S and SLS), respectively (Figure 4D). No statistical difference was found in brown adipose tissue weight of the S and SLS groups (P>0.05).

Plasma concentrations of glucose, triacylglycerol, and total cholesterol are shown in Table 1. Glycemia and triacylglycerolemia did not differ between groups (P>0.05). Exercise (E and SLE) caused a lower concentration in total cholesterol (6.5%) compared to the sedentary groups (P<0.05).

**Table 1:** Plasma concentration of glucose, total cholesterol, and triacylglycerol in the sedentary (S), exercise (E), small-litter sedentary (SLS), and small-litter exercise (SLE) groups.

Blood parameters (mg/dL)	S	E	SLS	SLE
Glucose	76.69±2.08	71.63±2.03	77.05±1.81	77.37±2.51
Cholesterol	94.52±0.91	88.40±1.57#	93.14 ±0.87	89.80±1.22#
Triacylglycerol	66.22±2.53	63.10±1.99	65.71±2.98	63.89±1.96

Data are reported as means±SE (n=8-10 rats/group). ^#^P<0.05, *vs* S and SLS groups (ANOVA).

**Figure 5:**
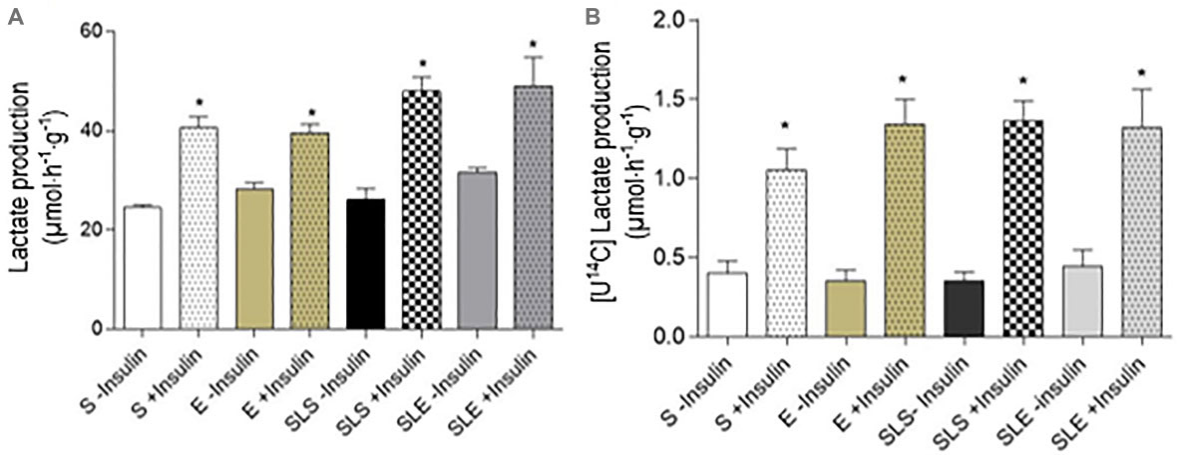
Total lactate (**A**) and radiochemical lactate (**B**) production from glucose in isolated incubated soleus muscles obtained from sedentary (S), exercise (E), small-litter sedentary (SLS), and small-litter exercise (SLE) rats. Muscles were incubated in Krebs-Ringer bicarbonate buffer, containing 5.6 mM glucose, 0.1 uCi/mL D-[U^14^C] glucose, for 1 h in the absence or presence of 10 mU/mL insulin. Data are reported as means±SE (n=8). *P<0.05 *vs* non-stimulated (ANOVA).

In basal condition (non-insulin), total lactate production (Figure 5A) did not differ between groups (P>0.05). The presence of insulin increased total lactate production by 1.7-fold in the S (P<0.003), 1.4-fold in the E (P<0.041), 1.8-fold in the SLS (P<0.0005), and 1.5-fold in the SLE (P<0.027 SLE+I *vs* SLE-I) groups. No statistical significance was found between insulin-stimulated groups (P>0.05).

Radiochemical lactate production (Figure 5B) in the absence of insulin did not differ between groups (P>0.05). The presence of insulin increased radiochemical lactate production by 2.7-fold in the S (P<0.0064), 3.8-fold in the E (P<0.0005), 4.0-fold in the SLS (P<0.0003), and 3.0-fold in the SLE (P<0.0055) groups. No statistical significance was found between insulin-stimulated groups (P>0.05).

**Figure 6:**
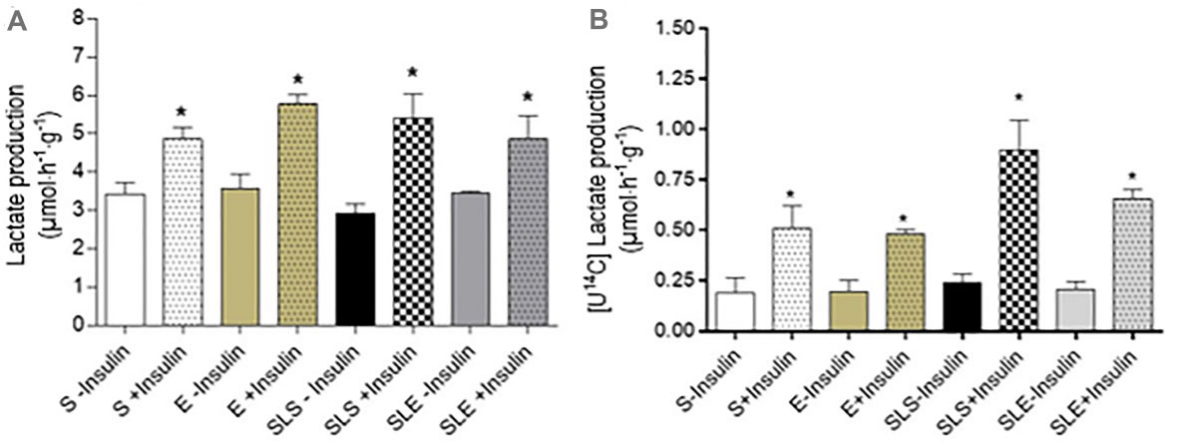
Total lactate (**A**) and radiochemical lactate (**B**) production from glucose by epididymal isolated incubated fat tissues obtained from sedentary (S), exercise (E), small-litter sedentary (SLS), and small-litter exercise (SLE) rats. Epididymal fat tissue was incubated in Krebs-Ringer bicarbonate buffer, containing 5.6 mM glucose, 0.1 uCi/mL D-[U^14^C] glucose, for 1 h in the absence or presence of 10 mU/mL insulin. Data are reported as means±SE (n=8). *P<0.05 *vs* non-stimulated (ANOVA).

Under non-stimulated conditions (Figure 6A), total lactate production by epididymal fat tissue did not differ between groups (P>0.05). In the presence of insulin, total lactate production by epididymal fat tissue was increased in the S and E groups (1.4-fold and 1.6-fold, respectively) compared to non-stimulated conditions (P<0.05). In SLS and SLE groups, insulin increased lactate production by 1.8-fold and 1.4-fold, respectively (P<0.05 *vs* non-insulin). Regarding [U^14^C]-lactate production (Figure 6B), no change was seen in the absence of insulin between groups (P>0.05). The presence of insulin increased the [U^14^C]-lactate production by 2.7-fold and 2.5-fold in the S and E groups, respectively (P<0.05). In the SLS and SLE groups, the increase was by 3.5-fold and 3.1-fold, respectively (P<0.05 *vs* non-stimulated).

**Figure 7:**
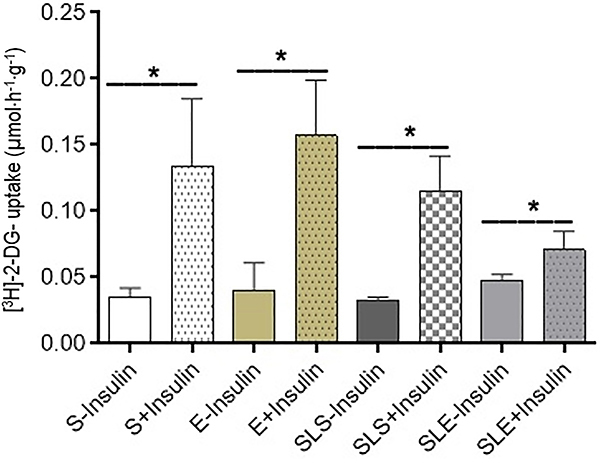
[^3^H]-2-deoxy-D-glucose (2DG) uptake by isolated incubated soleus muscles obtained from sedentary (S), exercise (E), small-litter sedentary (SLS), and small-litter exercise (SLE) rats. Muscles were incubated in Krebs-Ringer bicarbonate buffer, containing 5.6 mM glucose, 0.1 μCi/mL 2-DG, for 1 h in the absence or presence of 10 mU/mL insulin. Data are reported as means±SE (n=8). *P<0.05 (ANOVA).

[^3^H]-2-deoxy-D-glucose (2-DG) uptake by soleus muscle (Figure 7) in basal condition did not differ between groups (P>0.05). In the presence of insulin, the 2DG-glucose uptake by the soleus muscles of all groups increased compared to the basal condition (P<0.05 *vs* non-stimulated). The 2-DG uptake increased in the S and E groups by 3.9-fold. In the SLS and SLE groups, there was an increase of 1.7-fold and 1.5-fold, respectively.

## Discussion

Childhood and adolescent obesity is a major health concern and its prevalence has increased worldwide (20,21). Energy intake during lactation can induce alterations in the intermediary metabolism leading to life-long metabolic disturbances (4). Our results corroborate these findings, as 90-day-old rats raised in small litters had obesity. As the rats did not have hyperphagia, obesity was not caused by it. We know that before weaning, the newborn is supplied with nutrients exclusively through the mother's milk (22). An alteration in the quantity and quality of milk content might occur, and this may change the hypothalamic network involved in the energy homeostasis, ultimately leading to obesity (3,5,6).

In the SLS group, the body mass started to increase significantly from 60 days to 90 days of age, accompanied by insulin resistance, and higher mesenteric, epididymal, and retroperitoneal white fat mass without alteration in the brown adipose tissue (BAT) mass. Visceral fat has been associated with insulin resistance in rats submitted to nutritional interventions in gestation and lactation (15,16). Interestingly, despite insulin resistance *in vivo*, glycemia and triacylglycerolemia (TAG) were not changed in the SLS group nor in the other groups (P>0.05). On the other hand, the introduction of exercise reduced cholesterolemia (P<0.05 *vs* non-exercise groups). Insulin resistance, both in diabetic and nondiabetic subjects is associated with excess central obesity (23). The diagnosis of T2D is associated with insulin resistance and beta-cell dysfunction (24). It has been reported that nutritional intervention during fetal and early post-natal life affects beta-cell development and glucose-induced insulin secretion (25,26). Therefore, we hypothesize that despite insulin resistance, perhaps at this age, insulin secretion by beta cells is not impaired enough to change glucose and TAG concentrations. This hypothesis remains to be tested.

T2D, dyslipidemia, obesity, and insulin resistance are associated with low-grade chronic inflammation (20). How inflammation is triggered in obesity is not fully understood. One theory is that adipocyte hypertrophy and hyperplasia lead to the expansion of adipose tissue. As a consequence, large adipocytes will not get enough oxygen supply leading to hypoxia, which activates cellular stress pathways. The result is cell autonomous inflammation followed by the release of adipokines (resistin, adiponectin, leptin, among others) that might affect insulin sensitivity in the adipose tissue and skeletal muscle metabolism (27,28). Our approach of early introduction of exercise (starting at 22 days of age) did cause positive effects in rats. SLE rats did not increase body weight compared to the sedentary groups, and the brown fat pad increased significantly, accompanied by non-insulin resistance. During exercise, the tissues demand energy supplied by increased lipolysis and a reduction in the re-esterification of fatty acids followed by an increase in the fatty acid oxidation by peritoneal tissues and particularly by skeletal muscle, leading to a reduction in fat mass (29,30). The improvement in insulin sensitivity caused by fat mass reduction might be related to a change in the immunometabolism of adipose tissue due to exercise (31). It has been reported that exercise reduces the diameter of adipocytes, elevates the secretion of anti-inflammatory adipokines, and changes the phenotype of infiltrated cells in the adipose tissue (32). Exercise increases BAT metabolic activity and expansion (33). Exercise activates the sympathetic nervous system (34), which increases mitochondrial biogenesis and synthesis of uncoupling proteins that will cause an increase in fatty acid oxidation (35). The expansion of BAT might be related to a reduction of circulating lipids. We suggest that exercise affects BAT as an auxiliary mechanism to counterbalance fat mass gain. Pahlavani et al. (36) reported that obese animals have lower visceral adipose mass associated with higher BAT mass when fed with eicosapentaenoic acid. We found a similar result, but we used exercise instead of diet change.

Liver, SM, and AT are the target tissues of insulin (37). In SM, insulin stimulates glucose uptake by translocation of GLUT4 to plasma membrane (8). Therefore, impaired SM insulin signaling results in decreased glucose disposal. Once bound to its receptor, a complex downstream signaling cascade occurs that can be split into two main branches. One is the PI3K-AKT (also known as protein kinase B) pathway, widely responsible for insulin action on glucose uptake (27). The other branch is the Ras-mitogen-activated protein kinase (MAPK) pathway, which besides mediating gene expression also interacts with the PI3K-AKT pathway to control cell growth and differentiation. Both pathways have insulin-receptor-substrate (IRS) activation in common. There is also a non-IRS insulin signaling whose insulin mediator is the heterotrimeric G-protein. In AT, insulin signaling provokes a reduction in the activity of hormone-sensitive lipase, which inhibits free fatty acid (FFA) efflux from adipocytes. This is important because the increased concentration of FFA in the blood results in a reduction of insulin sensitivity and responsiveness in SM due to an elevation of lipid products within the cell, particularly ceramide and fatty acyl-coenzyme A (37). Consequently, these lipids activate the serine-threonine kinase PKC-θ, which, in turn, inhibits the insulin signaling cascade (27).

Obesity modifies carbohydrate (CHO) metabolism by insulin-dependent tissues, particularly skeletal muscle (9). We investigated *in vivo* and *in vitro* the CHO metabolism in adipose tissue and skeletal muscle. Obesity causes insulin resistance *in vivo*. The study of the glycolytic pathway with no interference of systemic factors is possible by the *in vitro* approach (29). SM and AT are the two main sites for glucose metabolism, and both are insulin-dependent for glucose uptake (7). In the SM and AT, the rate of glycolysis and 2-DG glucose uptake was not modified by obesity, meaning that once inside the cell, glucose is used as an energy source. In both insulin-dependent tissues, the presence of insulin increased total lactate and radiochemical lactate production as well 2-DG glucose uptake. Thus, *in vitro*, all groups presented a normal insulin-responsiveness. Therefore, the insulin resistance seen*in vivo*was probably caused by circulating factors. Hotamisligil et al. were the first to report the increased expression and production of TNF-α in adipose tissue of obese subjects and its key role in obesity-induced insulin resistance (38). In the last two decades, several reports corroborated this finding and demonstrated a marked infiltration of immune cells, especially macrophages in adipose tissue of rodents and humans (39,40). Therefore, dyslipidemia and pro-inflammatory cytokines seem to play an important role in insulin resistance.

In summary, our results suggested that the earlier exercise is started, the faster the harmful effects of premature obesity can be reduced or even stopped.
